# An Atypical Presentation of Acute Syphilitic Posterior Placoid Chorioretinitis With Concurrent Psoriasis Vulgaris Flare in an African American Male Infected With HIV

**DOI:** 10.7759/cureus.40294

**Published:** 2023-06-12

**Authors:** Matthew Melton, Alice Thornton, Sandro Pasagic

**Affiliations:** 1 Dermatology, University of Kentucky, Lexington, USA; 2 Infectious Disease, University of Kentucky, Lexington, USA; 3 Internal Medicine, University of Kentucky, Lexington, USA

**Keywords:** pathology derm, psoriasis, infectious disease, syphilis, dermatology

## Abstract

The diagnosis of secondary syphilis can be challenging due to its various clinical and histopathological presentations. A late or incorrect diagnosis can result in disease progression with consequent morbidity or mortality. Due to the importance of a correct diagnosis and specific treatment, it is of the utmost importance for healthcare providers to consider the various manifestations of syphilis. We describe an atypical presentation of secondary syphilis in an African American man infected with HIV and chronic psoriasis, who presented with two months of diffuse maculopapular rash and new visual changes, found to have acute syphilitic placoid chorioretinitis (ASPCC) and a psoriasis vulgaris flare.

## Introduction

There has been an increase in the incidence of syphilis over the last 10-15 years, coinciding with increasing transmission rates of HIV infection [1-2]. Syphilis is a sexually transmitted infection caused by *Treponema pallidum*; it is historically known as “The Great Imitator” due to its wide spectrum of disease manifestations and physical presentation. Due to the complexity of this disease, it is of utmost importance for healthcare providers to recognize the various presentations and be able to manage the infection properly, including avoiding steroids and other immune modulators that could result in further progression and spread of the infection. Syphilis manifestations can be further altered by comorbid conditions, such as HIV or psoriasis. Atypical syphilis cases have been described more frequently in patients with concomitant HIV infection [3-5]. Psoriasiform syphilis is an atypical presentation that is important to consider in patients with psoriasis on immunosuppressants. Another atypical presentation includes ocular syphilis with acute posterior placoid chorioretinitis (ASPPC). ASPPC describes the large, roundish, yellowish placoid lesion occurring at the level of the retinal pigment epithelium (RPE) at the macular/paramacular area [6]. Although this presentation is most likely to occur in secondary or tertiary syphilis, it can happen at any stage. In the acute phase, the uveitis is florid, progresses rapidly, and is often associated with meningeal involvement. Here, we describe a patient with HIV and psoriasis who had a rare and atypical presentation of syphilis that manifested only as a flare of psoriasis and decreased visual acuity.

## Case presentation

**Figure 1:**
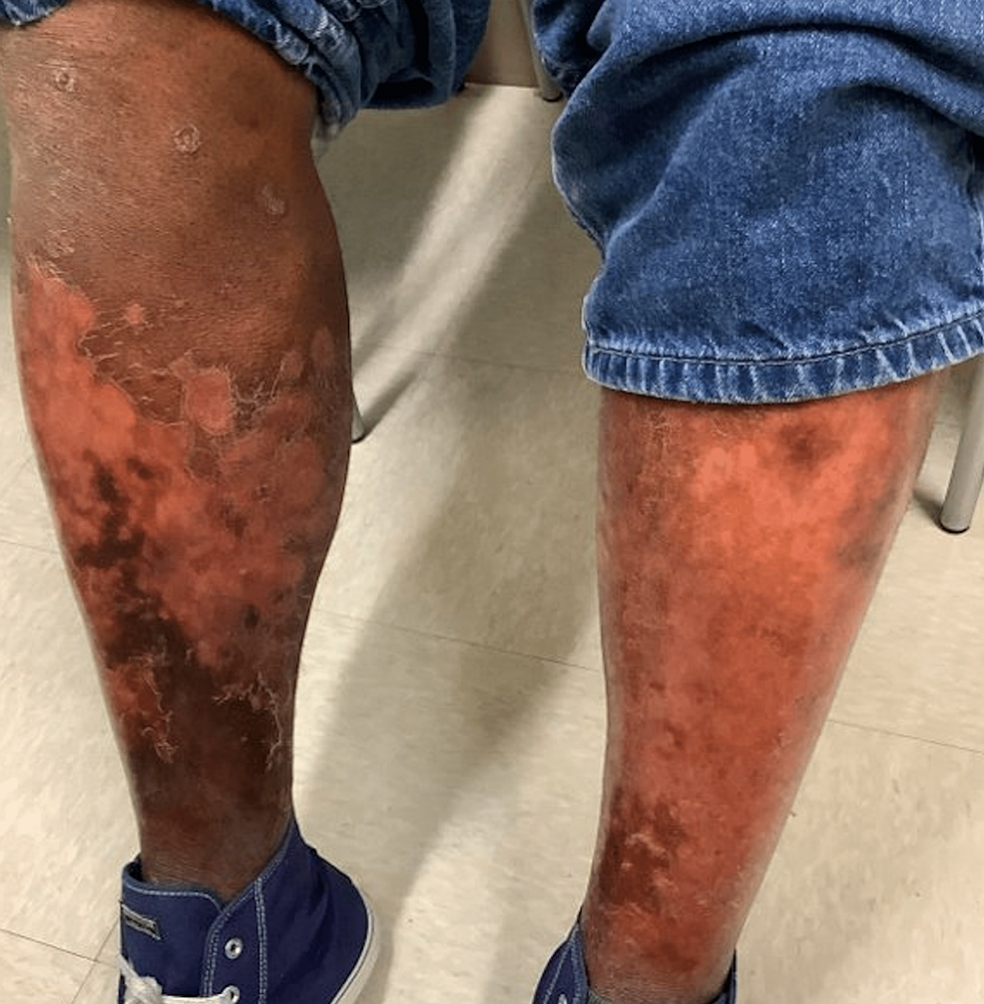
Initial presentation Initial presentation. Patient complaining of new-onset discoid rash with hypopigmentation, xeroderma, and erythema and worsening venous stasis rash on lower extremities​

A 52-year-old African American man who has sex with men presented to his PCP with a one-month history of pruritic, diffuse, and worsening maculopapular rash with plaques on the upper and lower extremities and back that spared the hands and feet, along with worsening stasis dermatitis (Figure 1). This patient has a significant history of congestive heart failure, atrial fibrillation, stasis dermatitis, hypertension, chronic kidney disease, gout, psoriasis, and well-controlled HIV with a viral load undetectable since 2015. Medications included elvitegravir/emtricitabine/tenofovir alafenamide/cobicistat (Genvoya®), apremilast, carvedilol, clobetasol cream, bumetanide, terazosin, and clindamycin/lincomycin cream. He had recently started apixaban and allopurinol just before symptom onset. Due to the recent changes in medications, an allopurinol drug reaction was suspected, including Drug Reaction with Eosinophilia and Systemic Symptoms (DRESS) syndrome or Stevens-Johnson syndrome (SJS). The patient was treated symptomatically with clobetasol cream and allopurinol cessation. He was seen by his dermatologist the following week, who also assumed his worsening psoriasis was secondary to a drug reaction.

**Figure 2:**
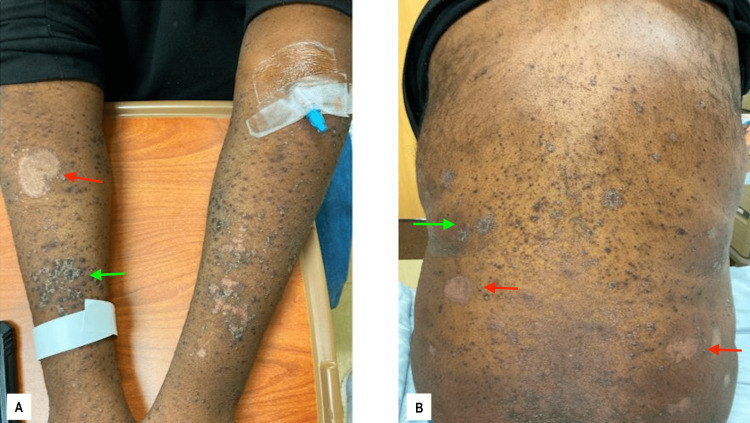
Second presentation Secondary presentation, one month later. The patient ​was now complaining of a progressive rash that spread ​to the arms (A) and back (B). Note the discoid lesions (red arrows) and​ the hyperpigmented, scaled papules (green arrows).

**Figure 3:**
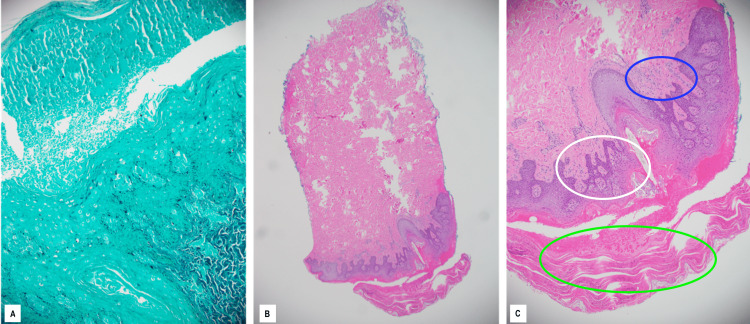
Punch biopsy of lesions on the back Punch biopsy of the inferior lumbar aspect of the back. ​GMS staining (A)​ Hematoxylin-eosin (H&E) staining; original magnification, ×40 (B) and ×100 (C) The GMS stain does not demonstrate fungal organisms. H&E stain shows the epidermis demonstrates psoriasiform hyperplasia (white circle, C) with a parakeratotic stratum corneum (green circle, C) with numerous polymorphonuclear cells (blue circle, C). The superficial viable epidermis demonstrates pallor and hypogranulosis. The superficial dermis has a mild perivascular lymphocytic inflammatory infiltrate.

The patient was admitted to the university hospital one month following the initial symptom onset with continued complaints of worsening pruritic maculopapular rash, new-onset right-sided blurred vision, and throat irritation (Figure 2). A dermal punch biopsy was obtained from the rash, which was initially unremarkable but was positive for *Staphylococcus aureus* at 24 hours. The patient was initially started on IV vancomycin for a possible bacterial skin infection. Ophthalmology was consulted for acute visual changes and found that the patient’s right eye vision was 20/200, decreased from baseline, with normal tonometry bilaterally. Ophthalmology determined there was posterior uveitis with placoid chorioretinitis and optic disc edema in the right eye. This was believed to be consistent with syphilis uveitis and the ASPCC. Despite having a negative RPR (rapid plasma regain) titer four months prior, RPR with Treponema pallidum antibody confirmed an acute active syphilis diagnosis with a titer of 1:256. The dermatopathologist described the initial skin biopsy as an epidermis demonstrating psoriasiform acanthosis with a parakeratotic stratum corneum with numerous polymorphonuclear cells. The superficial viable epidermis demonstrated pallor and hypogranulosis. The superficial dermis had a mild perivascular lymphocytic inflammatory infiltrate. The GMS stain did not demonstrate fungal organisms. Images of the biopsy are shown in Figure 3, demonstrating both Grocott-Gomori's methenamine silver stain (GMS) and hematoxylin/eosin stain.

**Figure 4:**
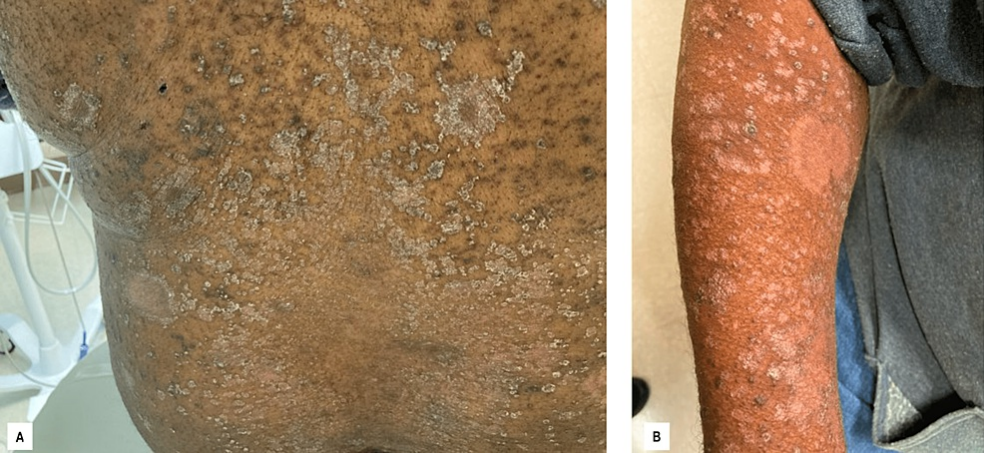
Follow-up evaluation Photos taken one week after discharge from hospitalization. ​Note the pallor located at healing lesions on the back (A) and arms (B). At this time, the​ patient claimed that the lesions were getting better and his symptoms were resolving.

A diagnosis of ocular syphilis with a psoriasis vulgaris flare was made. The patient deferred lumbar puncture evaluation as the treatment is identical for ocular syphilis and neurosyphilis. The patient was treated with 4,000,000 units of penicillin G IV every four hours, for a total of 32,000,000 units per day for 14 days. He was also given steroid eye drops, which improved his vision each day. On the fifth day, the patient reported feeling near baseline upon discharge. The patient was seen again for a follow-up four days after discharge, where the rash appeared to be resolving, and the vision continued to improve (Figure 4). The patient was seen again 14 days after discharge, where the rash continued to improve, his eyesight was continuing to return, and he returned to work. Two months after discharge, a repeat RPR was 1:64 with a nearly completely resolved rash. A four-fold decrease in RPR is denoted as a therapeutic response to therapy. The consulting dermatologist reiterated that this presentation was consistent with a psoriasis vulgaris flare, likely secondary to a recent syphilis infection.

## Discussion

A correct diagnosis was complicated in this patient with a well-controlled, undetectable HIV infection and additional confounding variables, such as a concurrent psoriasis diagnosis and dark skin [7,2]. Any rash on a sexually active person should cause concern for syphilis. Due to a history of psoriasis, it is easy to discount a rash. However, it is important to consider the cause of a psoriatic flare. HIV has also been implicated in psoriasis and can further complicate treatment. In this case, syphilis was acute because the patient had a negative RPR four months before the onset of symptoms. Unfortunately, syphilis was not considered in this patient, as the only presenting symptom was a psoriasis exacerbation. Interestingly, this patient was diagnosed with HIV in 2015 by dermatology, with the only presenting symptom being new-onset and severe plaque psoriasis. Psoriasis flares are a known phenomenon, often related to various infections, increased alcohol intake, or other stress-inducing disorders. *Streptococcus pyogenes*, which typically causes strep pharyngitis, is associated with guttate psoriatic flares [8]. Other associated infectious triggers have also been noted, such as viral infections like HPV and even common fungal infections like *candida* [9-11]. This can happen through multiple pathways, with some bacterial infections causing exacerbations via antigen activation of skin-seeking T cells [11].

In the clinical setting, the discovery of syphilis can often be difficult due to the heterogeneity of presentations and the presence of comorbid conditions, which may have disease-mimicking and modifying effects [5]. Syphilitic infections tend to occur more commonly in high-risk populations, such as men who have sex with men, sex workers, and injection drug users. These high-risk populations are more likely to have co-infections with other sexually transmitted infections, such as HIV [1]. An epidemiologic review of 30 studies found that the median seroprevalence of HIV in those infected with syphilis was 15.7% [12]. The immunosuppressive effects of HIV may decrease the likelihood of spontaneous clearance of syphilitic infections, further confounding and altering the typical course of a syphilis infection [5]. Our patient was on an apremilast immunosuppressant for his psoriasis, which could have also contributed to his atypical presentation. Immunosuppression can also result in an attenuated progression to neurosyphilis due to an impaired host response [5,13].

Some atypical syphilitic features that can present in HIV coinfection include ocular syphilis, multiple deep chancres with primary infection, and overlap of primary and secondary syphilis. Our patient did not have a chancre or other penile lesions, leading us to believe that this was not a primary syphilis infection. However, based on acute changes in vision, direct visualization of the posterior optic pole revealed a posterior macular plaque consistent with ASPCC. The placoid form of chorioretinitis is characterized by a ground-glass pattern, easily differentiated from the typical whitish necrotic lesions of both herpes and *Toxoplasma gondii* [14,15].

Once a diagnosis of syphilis was made, the medical team questioned if the worsening maculopapular rash represented a psoriasis exacerbation or psoriasiform syphilis. Although rare, there are several case reports describing an atypical cutaneous psoriasiform presentation of syphilis [3-4,7,16]. These cases typically present with scaly, erythematous plaques on the trunk or the palms and soles, resembling psoriasis. Basic histology is very similar to a biopsy performed for psoriasis. However, slight irregular acanthosis with elongated rete ridges and the presence of a consistent lymphoid infiltrate with numerous plasma cells, together with endothelial swelling, may point to irregularities that are more consistent with syphilis infection [11]. Patients are often initially misdiagnosed and treated with immunosuppressive therapies, such as corticosteroids. This results in lesion progression, which, in turn, often prompts further investigation that reveals a treponemal infection. After appropriate therapy, there is typically a complete resolution of psoriasiform lesions [11].

As mentioned prior, particular groups are at increased risk for syphilis infections due to high-risk behaviors or immunosuppression. Due to the variety of presentations that syphilis can manifest, clinicians need to be aware of atypical presentations and how syphilis can impact chronic conditions. It is also important to have a very high index of suspicion for acute symptoms, especially in these high-risk populations. Finally, routine surveillance with yearly RPR, yearly dilated eye exams, and patient education about warning signs are important for prompt recognition and treatment.

## Conclusions

In conclusion, this case highlights the importance of having a high clinical suspicion of syphilis in patients who present with a new-onset rash, particularly those who are immunosuppressed. This case also underscores the challenges of diagnosing syphilis, as the initial presentation did not fit with the typical symptoms of syphilis. 

The consequences of an undiagnosed syphilis infection can be severe and life-threatening. In this case, the patient's ocular syphilis could have led to permanent vision loss if left untreated. Additionally, syphilis can also lead to other serious complications, such as neurosyphilis and cardiovascular syphilis. That is why early diagnosis and prompt treatment of syphilis are crucial to preventing these negative outcomes. Clinicians should always consider syphilis in the differential diagnosis of patients with a new rash, particularly those who are immunosuppressed and should pursue appropriate testing to rule out the possibility of an acute or chronic syphilis infection. 
